# Tumour Heterogeneity and the Consequent Practical Challenges in the Management of Gastroenteropancreatic Neuroendocrine Neoplasms

**DOI:** 10.3390/cancers15061861

**Published:** 2023-03-20

**Authors:** Isabella Reccia, Madhava Pai, Jayant Kumar, Duncan Spalding, Andrea Frilling

**Affiliations:** 1 General Surgical and Oncology Unit, Policlinico San Pietro, Via Carlo Forlanini, 24036 Ponte San Pietro, Italy; 2 Division of Surgery, Department of Surgery & Cancer, Imperial College London, Hammersmith Hospital, Du Cane Road, London W12 0HS, UK

**Keywords:** neuroendocrine tumour, neuroendocrine neoplasms, gastroenteropancreatic, tumour heterogeneity, cell signalling pathway, biopsy, radioligand therapy, somatostatin analogues

## Abstract

**Simple Summary:**

Neuroendocrine tumours, recently reclassified as neuroendocrine neoplasms (NENs), are a heterogeneous group of tumours with variability in their disease course and outcome. Complex mechanisms involving spatial and temporal changes in tumour biology affect their treatment response and survival. Treatment strategies are often based on information regarding tumour stage and grade. Tumour heterogeneity, however, is a common phenomenon in NENs, and it is not uncommon for these neoplasms to show intra- and inter-tumour heterogeneity that may lead to incomplete understanding of their tumour biology and behaviour. This review summarises the available evidence on gastroenteropancreatic NEN heterogeneity and its impact on diagnosis and clinical management.

**Abstract:**

Tumour heterogeneity is a common phenomenon in neuroendocrine neoplasms (NENs) and a significant cause of treatment failure and disease progression. Genetic and epigenetic instability, along with proliferation of cancer stem cells and alterations in the tumour microenvironment, manifest as intra-tumoural variability in tumour biology in primary tumours and metastases. This may change over time, especially under selective pressure during treatment. The gastroenteropancreatic (GEP) tract is the most common site for NENs, and their diagnosis and treatment depends on the specific characteristics of the disease, in particular proliferation activity, expression of somatostatin receptors and grading. Somatostatin receptor expression has a major role in the diagnosis and treatment of GEP-NENs, while Ki-67 is also a valuable prognostic marker. Intra- and inter-tumour heterogeneity in GEP-NENS, however, may lead to inaccurate assessment of the disease and affect the reliability of the available diagnostic, prognostic and predictive tests. In this review, we summarise the current available evidence of the impact of tumour heterogeneity on tumour diagnosis and treatment of GEP-NENs. Understanding and accurately measuring tumour heterogeneity could better inform clinical decision making in NENs.

## 1. Introduction

Tumour heterogeneity refers to spatial and temporal variations that may occur within the tumour environment (intra-tumour) or within individual tumour foci, and also between tumour sites (inter-tumour) [1]. Such heterogeneity may encompass genetic and epigenetic variations, or differences in the tumour microenvironment [2,3,4,5,6]. Tumour heterogeneity can also evolve over time due to selective pressures, such as those imposed by treatment, leading to selection and clonal expansion of subpopulations [1,7,8,9]. Tumour heterogeneity is common in human tumours and its occurrence is essential to understand and predict tumour progression and response to specific treatment [10]. Higher intra-tumour and/or inter-tumour heterogeneity can be associated with negative outcomes [7].

Neuroendocrine tumours (NETs), better defined as neoplasms (NENs), are a heterogeneous group of neoplasms that range from well-differentiated tumours to more aggressive carcinomas (Table 1) [11].

The hallmark of NENs is their expression of somatostatin receptors (SSTRs), as somatostatin inhibits cell growth and hormone secretion in normal and cancerous neuroendocrine cells [12]. Somatostatin receptors are G-protein-coupled receptors with a typical transmembrane domain that includes five distinct subtypes named 1 to 5, with the gene encoding for the SSTR2 also producing two splice variants, SSTR2 isoform A and B. While the natural ligands of SSTRs (i.e., somatostatin-14, somatostatin-28 and cortistatin) all bind to the receptors with high affinity, somatostatin analogues (SSAs)-octreotide, vapreotide and lanreotide-bind only to SSTR2 and with a lower affinity to SSTR3 and 5 [13,14,15]. Neuroendocrine neoplasms express all SSTRs at different concentrations, with SSTR2 being the predominant receptor found across NENs of different origins, followed by SSTR3 in gastroenteropancreatic (GEP)-NENs and SSTR1 and SSTR5 in midgut NENs [16,17,18].

The GEP tract is the most common site for NENs, with the small intestine (SI) and the pancreas being the most prevalent sites of origin for more advanced neoplasms. For these neoplasms, treatment strategies are based on information on SSTRs expression, tumour stage and grade (including differentiation) and the expression of neuroendocrine biomarkers [19].

The definitive diagnosis of an NEN is made by histopathological examination of tumour tissue, obtained either via a biopsy or following surgery. Morphologic imaging, however, is essential as a baseline evaluation for staging, in particular for identifying the presence of metastases, while functional imaging is important to assess the functional and metabolic status of the tumour. Combining morphological (e.g., computer tomography-CT) and functional imaging techniques is fundamental in the decision-making process of the therapeutic approach to patients with GEP-NENs [20]. Gallium68 (68Ga)-DOTA-peptide positron emission tomography (PET)/CT, i.e., 68Ga-DOTATATE or 68Ga-DOTATOC, remains the gold standard for assessing the eligibility and response to peptide receptor radionuclide therapy (PRRT), especially for well-differentiated grade 1 and grade 2 GEP-NETs [21,22]. However, NENs often show heterogeneous expression of SSTR, which could lead to inferior outcomes following targeted treatment and subsequently influence relapse and progression of the disease [21,22,23,24,25]. High-grade lesions and metastases can have a lower expression of SSTRs which may not be fully assessed on receptor-based imaging alone.

Spatial and temporal heterogeneity should be taken into account in the assessment of NENs, as it is not uncommon for GEP primary and metastatic sites to show intra-tumour and inter-tumour heterogeneity in their Ki-67 index, as well as in their SSTR expression and cell signalling pathways, leading to incomplete understanding of their tumour biology and behaviour [26,27,28,29,30,31].

The aim of this review is to summarise the current available evidence on tumour heterogeneity in GEP-NENs (pancreatic and SI), and to evaluate the impact of this on clinical practice. We initially summarise the current available evidence on tumour heterogeneity in GEP-NENs. We then describe the manifestations of tumour heterogeneity and highlight their influence on diagnosis and clinical practice. Finally, we analyse the major problems limiting the efficacy of targeted strategies and affecting treatment outcomes.

Spatial and temporal heterogeneous expression of SSTRs, Ki-67 and cell signalling pathways can be used to predict tumour progression, prognosis and response to targeted therapeutic strategies [21,26,32]. Understanding of target heterogeneity in NENs could inform the selection of the best candidates for specific treatment, in order to predict response to treatment and prognosis and to plan appropriate management and follow-up.

## 2. Tumour Heterogeneity in GEP-NENs

**Figure 1:**
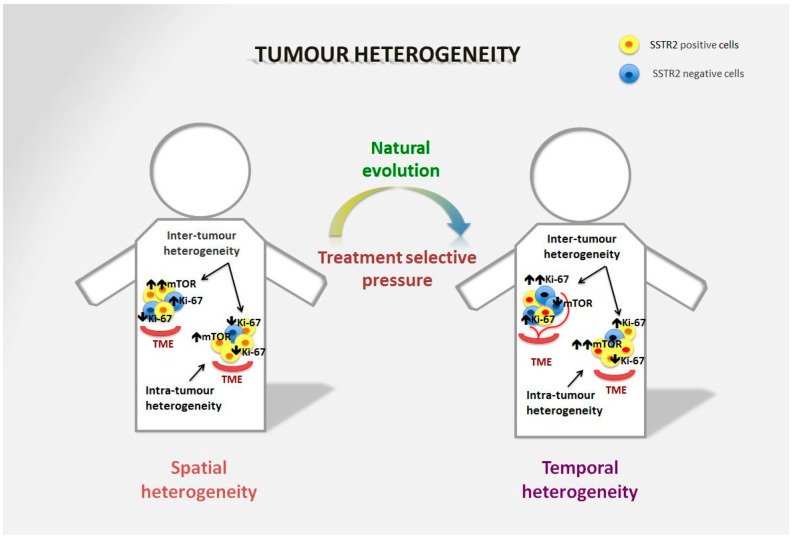
Spatial and temporal heterogeneity in NENs. Neuroendocrine neoplasms generally express SSTR2 on the tumour surface, and are well-differentiated tumours in the majority of cases. However, spatial heterogeneity within the primary tumour may lead to the presence of areas with lower expression of SSTR2 and/or a different Ki-67 index. This heterogeneity is also frequent in metastatic sites and can differ significantly from the primary lesion. The mTOR pathway is also commonly involved in the onset of the disease and is particularly relevant in distant metastases, although over time alternative pathways may be involved in tumour survival. Moreover, temporal heterogeneity that can be linked to treatment selective pressure may lead to significant changes in tumour biology that affect prognosis and survival.

Tumour heterogeneity is a common phenomenon in GEP-NENs (Figure 1) and has a negative impact on treatment success and prognosis as it produces cell clones that do not express treatment targets (i.e., SSTR, mammalian target of rapamycin–mTOR- signalling pathway, Ki-67) [33].

Pancreatic NENs can show a progressive increase in their Ki-67 index or progression to a more aggressive disease, events that are linked to poorer prognosis [34,35]. Changes in the intra-tumoural distribution of Ki-67 in GEP-NENs can lead to significant downgrading of tumours as a consequence of sample bias, especially when small samples are collected that include areas of non-neoplastic tissue [31,36,37]. The Ki-67 is one of the prognostic markers for NENs; however, evaluation of the Ki-67 depends on the site and size of the tumour biopsy and assessment by the pathologist, therefore it may be not representative of tumour behaviour in heterogeneous lesions and especially in intermediate grade 2 lesions [26].

Small-intestine NENs are generally considered to have a relatively low somatic mutation rate, but a more florid epigenetic derangement. It has been shown, however, that there is a high degree of genetic variability between the primary site and liver metastases [38]. Although they are generally well-differentiated tumours with low proliferation rates, distant metastases, in particular hepatic, are a common event and an important cause of poor prognosis [39,40]. The rate of mutations is high, especially in liver metastases, with the mutations often being different to the mutations seen within the primary tumour, thus demonstrating a unique pattern of metastatic spread of SI-NENs [38,41,42]. A large molecular profiling study on SI neuroendocrine liver metastases showed that the expression of several cancer-related pathways that promote tumour development, progression and angiogenesis, including phosphoinositide 3 kinase (PI3K), epidermal growth factor receptor (ErbB1), platelet-derived growth factor receptor beta (PDGFRβ) and mTOR, is upregulated in neuroendocrine liver metastases in comparison to their primary site, and that neuroendocrine liver metastases harbour progressive genomic aberrations that occur mostly during the metastatic progression of the tumour [43]. It has also been shown that the pattern of metastatic growth within the liver may be the expression of the different biological behaviour of the disease, as less-differentiated NENs more often showed an aggressive pattern of growth (disseminated metastatic spread) linked to higher Ki-67 and more advanced disease [44]. Most metastases of GEP-NENs show a higher Ki-67 proliferation index than the primary tumour site, meaning that metastatic spread is potentially unrelated to its initial phenotype or genotype [26,45,46]. SI-NENs are usually believed to display an even expression of SSTR2 isoform A [24]. However, SI neuroendocrine liver metastases often show heterogeneous SSTR2 isoform A expression between lesions in the same patient, this seems to be unrelated to the tumour proliferation index or the tumour size, confirming that expression in metastatic lesions is not always similar to that in the primary tumour or between lesions in the same patient [24,31]. Moreover, no correlation has been shown with the SSTR2 isoform A expression of the primary tumours [24]. Liver metastases from ileal NENs have also been found to show a higher expression of SSTR5, which is potentially linked to tumour aggressiveness [32]. Somatostatin receptor type 5 expression seems to correlate with the presence of metastases and angioinvasion in NENs [47]. Although imaging, in particular 68Ga-DOTATATE PET/CT scans, seems to detect most liver metastases even when SSTRs expression is weak, response to radioligand therapy (RLT) may be lower and different between lesions in the same patient [24,48]. Intra-tumour, and especially inter-tumour, heterogeneity should therefore be taken into account in the diagnosis and management of GEP-NENs, as it represents a major challenge for the efficacy of targeted therapies. A better understanding of tumour biology will help in maximizing treatment outcomes.

## 3. Morphologic Heterogeneity in GEP-NENs

Somatostatin receptor imaging is essential for the detection and characterisation of GEP-NENs, and adequate uptake is an important requirement for the use of SSAs and PRRT. The expression of SSTR2 is an independent positive prognostic factor for survival being linked to a longer overall survival following PRRT. Conversely, SSTR5 expression in GEP-NENs seems to be related to angioinvasion and metastases [32,47,49,50,51]. Existing data, however, varies greatly [13]. Of interest, NENs of the same origin can express different types of SSTRs, and SSTR heterogeneity is also a frequent event within the same patient, as demonstrated by Graf and colleagues, who found that more than 40% of patients with grade 1 and 2 NETs had heterogeneous inter-tumour SSTR expression at baseline imaging prior to PRRT [21]. The expression of SSTRs on imaging could therefore be more accurate in the assessment of tumour heterogeneity in comparison to tumour sampling (i.e., assessment of Ki-67 on tumour biopsy), as imaging can assess all primary and secondary lesions (spatial and morphological heterogeneity), and can be repeated to assess changes in tumour biology over time (temporal heterogeneity) [52]. Pre-treatment 68Ga-DOTA-peptides PET/CT, however, does not always correlate with a response to PRRT, as target lesion heterogeneity may affect the response [53]. The quality of SSTR expression on baseline 68Ga-DOTA-peptides PET/CT prior to PRRT has been proposed as a parameter to evaluate intra-tumour heterogeneity in grade 1 and grade 2 NETs. It has been shown that high tumour heterogeneity of SSTR expression on 68Ga-DOTA-peptides PET/CT (≥ 50% of patient’s lesions) is linked to shorter time-to-progression in comparison with homogeneous SSTR expression [21]. The quality of SSTR expression, however, was only assessed for clearly visible lesions with a size of 2 cm or larger on a CT scan in patients with well-differentiated NENs. In addition, SSTR2 expression decreases with increasing malignancy and tumour stage [54,55,56]. It has also been shown that the heterogeneous SSTR2 expression seen on 68Ga-DOTATOC PET/CT differed depending on the primary and metastatic sites, and that the coefficient of variation (CoV) was higher in NEN metastases than in the primary tumours [57]. The standardised uptake values (SUVs), in particular SUVmax (which expresses the SUV in a single pixel with the highest value within a region of interest), are semiquantitative measurements of uptake in cancer tissue [58]. There appears to be a link between SUVmax and the proliferative activity of GEP-NENs, as higher SUVmax values on 68Ga-DOTA-peptides PET/CT have been found in well-differentiated GEP-NENs (in particular pancreatic) which correlate with a better prognosis, while higher-grade GEP-NETs showed lower SUVmax values [59,60,61]. Others, however, have argued that there is no clear correlation between SUV values and outcomes [62,63,64]. SUVmax on 68Ga-DOTATATE imaging has been found to be higher in patients with bone metastases from NENs of different sites; generally bone metastases in NENs represent a poor prognostic factor [65]. The inconsistent correlation between SUV changes and outcomes could be due to the presence of spatial and temporal SSTR heterogeneity that may lead to a divergent appearance after treatment on morphologic (CT or magnetic resonance imaging-MRI) versus receptor-based imaging [53].

**Figure 2:**
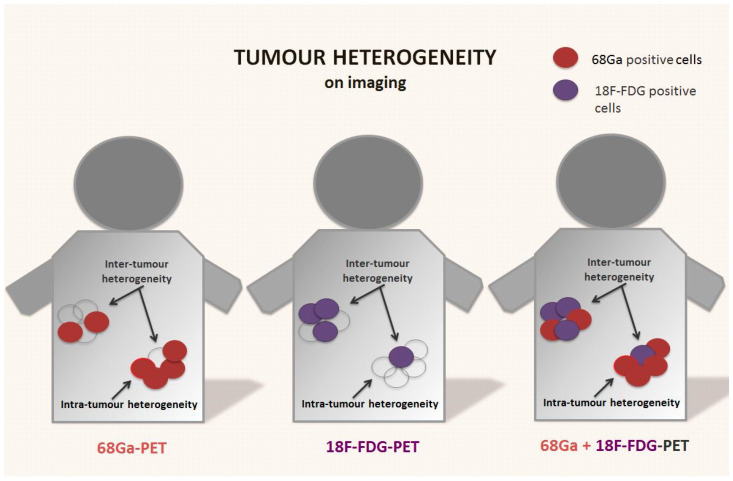
Tumour heterogeneity on receptor-based and functional imaging of NENs. Neuroendocrine neoplasms are usually positive on somatostatin receptor-based imaging (i.e., 68Ga-DOTA-peptides PET). However, the presence of tumour heterogeneity with areas that express lower or no somatostatin receptors is common on baseline scans, and often after disease relapse or progression. These areas are often less differentiated and metabolically active. The use of 18F-FDG-PET, especially in combination with receptor-based imaging, can potentially provide a better picture of metabolic spatial intra- and inter-tumour heterogeneity of the disease, especially in higher-grade tumours.

In less differentiated NENs that often co-express SSTRs and glucose-transporters, the addition of flourine-18 fluorodeoxyglucose (18F-FDG)-PET imaging can potentially better assess the intra- and inter-lesional heterogeneity of the tumour by exploring the heterogeneous distribution and uptake of 18F-FDG (Figure 2) [52].

18F-FDG-PET might be a useful addition to SSTR-based imaging in NENs with higher mitotic rates but well-differentiated morphology, i.e., for grade 3 GEP-NETs [66]. They might help in predicting response to PRRT, as standardised uptake values on 18F-FDG-PET seem to correlate more clearly than 68Ga-DOTA-peptides PET/CT with higher histological grades and poorer overall survival [53,67,68,69,70,71,72,73]. In a retrospective analysis of grade 3 GEP-NETs, patients who showed increased 18F-FDG uptake in comparison to 68Ga-DOTATOC had significantly lower survival than patients who displayed increased uptake with receptor-based imaging. Moreover, homogeneous uptake on 68Ga-DOTATOC correlated with a better overall survival in comparison to negative or inhomogeneous uptake, while negative or inhomogeneous uptake on 18F-FDG was associated with a poorer outcome in comparison to patients showing homogenous uptake on functional imaging [72,74]. Indeed, 18F-FDG-PET positive NENs that display low avidity on SSTR imaging are potentially high-grade, metabolically active and aggressive tumours with more rapid progression of the disease and a worse prognosis [75,76,77]. As interpretation of dual modality imaging in multifocal and heterogeneous tumours can be difficult, Chan et al. proposed a scoring system (the NETPET Grading Scheme) for metastatic NENs that combines information obtained by SSTR and 18F-FDG-PET that correlates with overall survival [77]. Similarly, Karfis et al. classified patients with metastatic GEP-NENs based on the predominant imaging uptake on 68Ga-DOTATATE and 18F-FDG PET/CT. They observed a significant difference in progression-free survival that correlated with heterogeneity of uptake on dual tracer imaging, as patients with GEP-NENs with homogeneous uptake on 68Ga-DOTATAE but at least one 18F-FDG positive lesion had better progression-free survival in comparison with patients with one or more 18F-FDG-positive lesions and at least one 68Ga-DOTATATE-negative lesion [78]. This difference was not shown when only the Ki-67 histological grade was taken into account. The European Neuroendocrine Tumour Society (ENETS) guidelines recommend the use of 18F-FDG PET/CT only for high-grade (grade 3) tumours, while somatostatin receptor imaging is indicated for well-differentiated (grade 1 and grade 2) NETs [79]. Although the routine use of 18F-FDG PET/CT in all grades of NENs cannot be justified in view of increased costs, it might be reasonable to suggest the addition of 18F-FDG PET/CT in those particular cases (i.e., intermediate Ki-67 index, high grade lesions on biopsy, heterogeneous or low uptake on somatostatin receptor imaging, clinically aggressive disease, poor response to PRRT) where high intra- and inter-tumour heterogeneity may not be revealed on only receptor-based imaging [80,81]. Dual imaging may give a better picture of intra-tumour heterogeneity in tumour biology (grading and SSTR expression) and guide biopsy and targeted therapeutic approach in NEN patients [46]. Dual imaging can also reveal synchronous heterogeneity, where some lesions in the same patient are FDG positive but 68Ga negative or vice versa. It may be particularly useful during surveillance, as receptor-based imaging alone may fail to show the change in tumour behaviour over time [76]. Finally, dual imaging could also be useful in detecting bone metastases that can be missed on 18F-FDG/PET alone in less differentiated GEP-NENs, as 68Ga-DOTATATE seems to be more accurate in detecting bone metastases regardless of the differentiation of the primary GEP-NEN [82].

## 4. Tumour Tissue Heterogeneity and the Impact on Biopsy

In NENs, random biopsies may not be representative of the whole tumour burden, especially for higher-grade tumours and metastatic disease, which often display a different clinic-pathological pattern from their primary site. More than half of metastatic GEPs and SI-NENs show a higher Ki-67 index on definitive histology and a subsequent change in World Health Organization grade [26,27,28,29,30,31]. There has been controversy over the accuracy of NEN biopsies, as it can be difficult to assess the complexity of the disease on cytological or small tissue samples [83,84]. The assessment of grading by the Ki-67 index can be affected by the size and type of tumour sample, with discordance between biopsy results and definitive histology and the risk of misinterpretation of tumour behaviour [85]. Moreover, it has also been debated that with grade 2 NETs, the tissue sample size and heterogeneity in the intra-tumour distribution of Ki-67 could negatively impact biopsy accuracy in GEP-NENs, as higher-grade tumours show significant clinical and molecular heterogeneity [27,36,86].

Tissue sample requirement (i.e., size and cellularity) and sampling modality are important factors in ensuring reliable results. Most of the evidence available is on pancreatic NENs, as primary SI-NENs are often inaccessible to biopsy, forceps biopsy is technically challenging for lesions located deep in the mucosa or sub-mucosa, and there is no validated cytology reporting system [87,88,89]. Several studies have assessed the reliability of cytology for the diagnosis of pancreatic NENs. Some authors have found that cytology failed to accurately assess tumour grade as a different Ki-67 index was found on the resected specimen, while others have shown that preoperative cytology assessment of the Ki-67 proliferation index was reliable especially for either low-grade or high-grade pancreatic NENs [90,91,92,93,94,95]. Intermediate proliferating NENs, however, are often misinterpreted, with the distinction between grade 1 and grade 2 tumours often not being possible. For pancreatic-NENs, concordance between fine-needle aspiration (FNA) cytology and the final histology following surgery has been reported to range between being very low to very high level. Among the studies, higher levels of concordance have been achieved when only samples with high cellularity were included, when the highest Ki-67 index was considered for grading, and for small lesions of less than 2 cm in diameter. Again, most pitfalls have been found for grade 2 tumours, which are often downstaged [83,96,97,98,99,100,101,102]. An adequate sample size using a needle that allow a total length of the sample of at least 15 mm with prevalence of neoplastic tissue (>80%) should help in minimizing the risks of misinterpretation of tumour grade [36]. A high level of suspicion for more aggressive disease should be maintained when there is discrepancy in cases where a low-grade lesion has been detected on biopsy for a disease that shows a more aggressive clinical and radiological behaviour.

For neuroendocrine liver metastases, FNA has been used to confirm diagnosis and to assess tumour grading [103,104]. In view of the increased intra- and inter-tumour heterogeneity in neuroendocrine liver metastases and the differences often seen in the primary tumour, however, biopsy is recommended, with a core needle or surgical biopsy still being the preferred modalities, as differentiation and grading have significant prognostic value in metastatic disease [26,105,106]. Nevertheless, it should be kept in mind that preoperative biopsies, both FNA and core, could wrongly assess grading in as many as a third of cases when compared with the grading seen on the surgical specimen of GEP neuroendocrine liver metastases. A repeat sample is necessary if incongruity is noted with the other information available [45,107].

**Figure 3:**
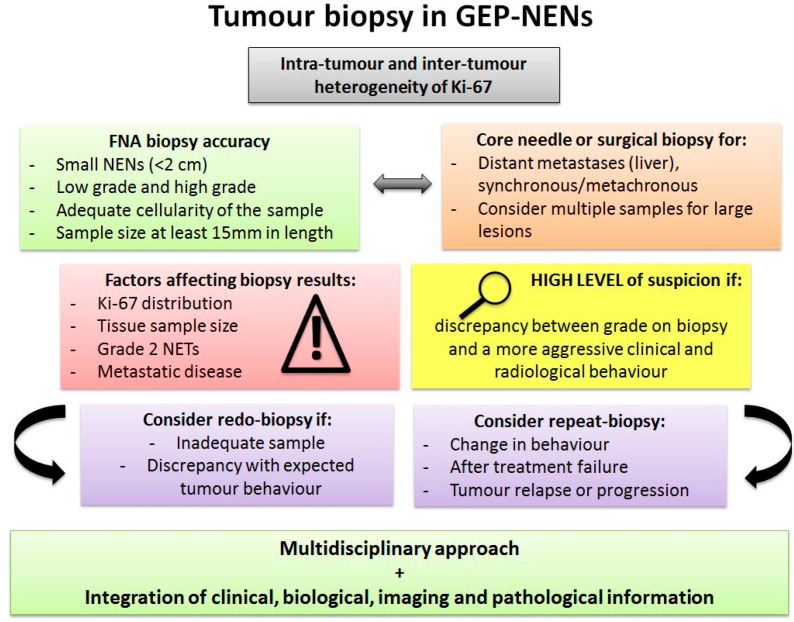
Tumour biopsy in GEP-NENs. Pitfalls and suggestions for tumour biopsy in GEP-NENs. Abbreviations: GEP: gastroenteropancreatic; FNA: fine-needle aspiration; NET: neuroendocrine tumour.

As NENs are often multifocal, and as there is often inter-tumour heterogeneity between primary and metastatic disease, the need for multi-regional and multi-site biopsies needs to be raised, as a single tumour-biopsy may be inadequate to assess the mutational landscape of NENs [108,109,110,111]. If it is agreed that primary and synchronous/metachronous metastases should be biopsied in light of what has already been discussed, the number of samples required remains unclear. It has been shown that there is frequently a discrepancy between grading on preoperative biopsies and the final histology for SI neuroendocrine liver metastases, suggesting that multiple biopsies may be necessary [107]. It has also been suggested that the largest lesion should be biopsied, as there seems to be a correlation between tumour size and tumour grade. As the largest lesions are also associated with higher spatial heterogeneity, however, multiple samples may be needed [31]. Nevertheless, multifocality is common even in well-differentiated SI-NENs, and does not seem to affect prognosis; therefore, biopsy of the largest lesion alone should be adequate to assess the tumour grading of the disease [112]. On the other hand, although multiple samples could theoretically be taken from a resected specimen, it may not be safe or recommended to perform multiple percutaneous or endoscopic biopsies as a biopsy is an invasive method and may be technically challenging depending on the site of the lesion [113]. While it is difficult to propose clear-cut guidance, as one size does not fit all in GEP-NENs, there is enough evidence to recommend biopsy of the primary and of metastatic disease, especially when metachronous, and that the need for multi-regional and multi-site biopsy should be based on the global picture of the disease, i.e., imaging, clinical course, and expected behaviour. The current guidelines also recommend reassessment of the Ki-67 when there is change over time in the clinical course of the disease [114,115]. The initial assessment of tumour tissue at the time of diagnosis or treatment does not reflect the changes that the tumour undergoes over time under the influence of the microenvironment and treatment selective pressure [116]. The assessment of the proliferative index on tumour biopsy after curative surgery from recurrent NENs of various sites, in particular GEP-NENs, has been shown to depict a higher proliferation index than the primary disease. Treatment itself can induce topographic heterogeneity in the Ki-67 index, with either a lower or higher heterogeneous expression of the Ki-67, suggesting that a repeat tumour biopsy, which is not routinely performed, might be needed [117,118,119]. Figure 3 summarises recommendations for NEN biopsies taking tumour heterogeneity into consideration (Figure 3).

In light of the complexity of NENs, a multidisciplinary approach and the integration of clinical, biological, imaging and pathological information are essential and fundamental to guide a tailored approach. Receptor-based imaging has been shown to correlate with the disease phenotype and the expression of SSTRs, as in most cases the high expression of SSRT is linked to a well-differentiated tumour. Positive 18F-FDG-PET uptake seems to correlate well with the Ki-67 index and the presence of tumour heterogeneity. In particular, 18F-FDG-PET might be helpful in the selection of target lesions to biopsy in other types of NENs [120,121,122,123]. Although the available evidence is still scarce, it also seems that CT ratio in dynamic CT may correlate with pathological grade in pancreatic NENs, and may be useful when adequate sampling for the assessment of tumour grading is not possible [124].

## 5. Radioligand Therapy

Peptide receptor radionuclide therapy is mainly used as second-line therapy for metastatic, inoperable, or progressive well to moderately differentiated NENs that originate from the GEP tract [19,125,126,127]. The effectiveness of PRRT in terms of progression-free survival in the management of NENs has been proven by a prospective randomised controlled clinical trial [128,129]. Current guidelines recommend PRRT treatment for well-differentiated NENs with positive receptor-based imaging that are locally advanced, progressive, unresectable or metastatic [19,127,130].

Responses to PRRT are unpredictable [131]. Among the factors that influence treatment response, the degree of SSTR2 expression of tumour cells at SSTR scintigraphy (i.e., Krenning score of 3 and 4) is critical, along with the site of the primary tumour, tumour burden, tumour grading, and imaging uptake on PET scan [132]. While 68Ga uptake is one of the criteria for the selection of suitable candidates for PRRT, it does not always correlate with PRRT efficacy, especially for NENs that lose SSTR expression [132,133]. Indeed, not all patients respond to PRRT, even though they appear to be good candidates on imaging. The effectiveness of PRRT depends on receptor expression; a poor response to PRRT may be due to the presence of intra-tumour heterogeneity with areas of low or no uptake that cannot be targeted by the radioligands. In vitro models and human samples from NENs expressing SSTR2 have shown that the DNA damage and apoptosis induced during PRRT are heterogeneous and significantly correlated with SSTR2 expression, with higher damage observed in areas of the tumour with a high expression of the target receptor, this was not explained by differences in proliferation nor in bioavailability of the radioligand [48]. The DNA damage response induced following Lutetium-177 (177Lu)-DOTATATE treatment has been visualised on imaging to demonstrate the impact of tumour heterogeneity of the biological response to PRRT in these models [134].

The use of 18F-FDG PET in the assessment of tumour progression, survival and the response to PRRT has shown a predictive value in patients with advanced NENs, thus 18F-FDG uptake could be more useful for high-grade NENs that often lose SSTR expression [53,132]. Although 18F-FDG PET has better spatial resolution and sensitivity in comparison to single photon emission computed tomography (SPECT), it has been shown that spatial heterogeneity can be demonstrated on SPECT when combined with CT imaging in well-differentiated metastatic GEP-NENs that show positive SSTR uptake on pre-PRRT Octreoscan. Indeed, patients who demonstrated a poor morphological response on 177Lu-SPECT/CT acquired at 24 h post injection during the first cycle of PRRT had significantly higher spatial heterogeneity in comparison to patients with a good response to treatment [135]. The asphericity, which expresses the shape irregularity of the functional lesion volume on SSRT imaging, may have a prognostic value in NENs receiving PRRT [69]. Asphericity could represent a method to quantify the spatial SSTR heterogeneity in NENs [136,137]. Moreover, since the uptake of tracers or therapeutic molecules is dependent on tumour vascularization, the combination of SPECT with MRI could predict peptide uptake more accurately then SPECT-CT scan [138]. Magnetic resonance imaging, in particular dynamic contrast-enhanced MRI (DCE-MRI) could be a valuable tool in highlighting tumour heterogeneity such as microvascular heterogeneity, characteristics of the microenvironment, and in predicting treatment response [139,140,141].

Patients with insufficient tumour uptake on SSTR imaging seem to have worse outcomes and a much lower survival even when receiving more aggressive treatment. This could be linked to an epigenetic gene silencing associated with abnormal tumour growth [142]. Of note, a number of liver metastases from SI-NENs may display a weak expression of SSTR2 at imaging that affects responsiveness to treatment [24]. Moreover, heterogeneous expression of SSTRs on target lesions of patients who have received PRRT has been linked to a lower overall survival and earlier progression, showing that SSTR heterogeneity is a strong predictive and prognostic factor in grade 1 and 2 NET patients who receive PRRT [21]. Indeed, patients with grade 1 and grade 2 NETs who have disease progression after PRRT have significant intra- and inter-lesional SSTR heterogeneity as demonstrated on 68Ga-DOTA-peptides PET scan. Grading does not always correlate with SSTR expression, as grade 3 liver metastases from well-differentiated NENs still display moderate to high SSTR expression at a level that allows PRRT to potentially work [24]. Grade 3 NETs are indeed highly heterogeneous in terms of SSTR expression and proliferation, and recent evidence suggests that PRRT should also be considered in grade 3 GEP-NETs that display sufficient uptake on receptor-based imaging as well as in cases with a Ki-67 index below 55% [143,144,145,146]. This cut-off seems to be the most accurate value to differentiate between grade 3 NETs and grade 3 neuroendocrine carcinomas (NEC) [147].

Temporal heterogeneity can be a cause of progression or relapse after PRRT as the majority of patients with initially stable disease, despite initial high disease control rates, will eventually progress [148,149,150,151]. Early progression and a shorter progression-free interval after PRRT could be due to intra-tumour- heterogeneity with the coexistence of low- and high-grade cell subtypes with the more aggressive subtype progressing more rapidly during PRRT that targets only the low aggressive subtype. Although the evidence is still limited, a repeat biopsy in a small group of patients who have rapid disease progression post-PRRT showed a more aggressive tumour than the initial histology [152].

## 6. Somatostatin Analogues Therapy

Somatostatin analogue therapy for NENs has been used to control symptoms in patients with functioning GEP-NENs since the late 1980s, and its use has subsequently been extended to treat all symptomatic NENs [153]. Current guidelines recommend the use of SSAs (octreotide, lanreotide) as a first-line choice in functionally active NENs such as vipomas and glucagonomas and for the treatment of carcinoid syndrome and prevention of a carcinoid crisis [19]. While the initial goal of SSA treatment was symptom control, subsequent evidence has demonstrated that SSAs are also able to inhibit tumour growth in selected patients with metastatic NENs [154,155]. The mechanisms for the antiproliferative effect of SSAs seem to be related to the activation of SSTRs, leading to induction of G1 cell cycle arrest and apoptosis, particularly via SSTR2, and to the inhibition of tumour angiogenesis and mitogenic circulating growth factors, although the antiproliferative effect of SSAs does not impact overall survival [156].

Well-differentiated SI and pancreatic NENs generally express high levels of SSTR2, especially in tumours with a low Ki-67 index (≤2%) [18,50,157]. Hence, SSAs are now used as a treatment option for patients with progressive well-differentiated pancreatic and midgut NENs, preferably with a Ki-67 of ≤10%, regardless of the functional status of the tumour, to either prevent or inhibit tumour growth [158]. First-generation SSAs, octreotide and lanreotide, the only two SSAs approved for NENs, bind specifically to SSTR2 and with lesser affinity to SSTR5, with lower to no affinity for the other SSTRs, while the new SSA pasireotide is also able to bind to the other SSTRs with greater affinity [12]. The effect of octreotide seems to be mediated mainly by the interaction with SSTR2, and SSTR2 is the target for receptor-based imaging and RLT in NENs. It has been shown that SSTR2 expression in pancreatic and SI-NENs correlate with a better prognosis and longer overall survival and that the benefits of SSA therapy on progression-free and overall survival are linked to the positive SSTR status of the tumour [50,142,159]. While SSTR2 expression is used to predict response to SSAs, the correlation between the pattern of expression of SSTR2 on tumour cells and the therapeutic response to SSAs is unclear as the available evidence is limited to only a few selected cases. In one study, the efficacy of SSAs was assessed in 14 gastrointestinal NEN cases and evaluated with regards to the SSTR2 immunoreactivity quantified with a different scoring system at histology. While SSTR2-positive cases were linked to either stable disease or a complete response after SSAs regardless of the scoring system used, half of the cases that were SSTR2 negative, according to one of the scoring systems used, also showed a response to SSAs. No statistically significant differences were noted [160]. Conversely, in another study on 12 patients with advanced non-functioning pancreatic NECs, response to SSAs was not related to the SSTR2 expression assessed by quantitative reverse transcription PCR [161].

Intra-tumour and inter-tumour (between primary and metastatic disease) heterogeneity in the expression of SSTR2 in NENs has also been demonstrated [24,48]. Inter-tumour heterogeneity of SSTR2 expression is frequent, especially in metastatic deposits, as in SI metastatic NENs it is common to find that at least one of the liver lesions, especially in larger lesions, shows either low or no expression at all of SSTR2 despite the rest of the tumour burden showing SSTR2 expression [23,24]. The impact of heterogeneous expression of SSTR2 on the efficacy of SSAs, however, is less clear. It is difficult to compare available evidence and clearly delineate whether the response to SSAs is truly linked to the expression of SSTR2. This is because case-series are small, the methods used to quantify SSTR2 expression differ, and the patients included are extremely heterogeneous in terms of disease and treatment received, reflecting the nature of NENs. Moreover, it is unclear whether there is an association between SSTR2 imaging and an effective response to SSAs, as it is possible that the presence of even a few receptors may in fact lead to a response to SSAs that affects the whole tumour burden. Temporal heterogeneity may also significantly affect the response to SSAs to a degree higher than the available evidence might prove. It is known that a response to treatment is inversely correlated with the length of treatment and development of treatment resistance is possibly linked to downregulation of SSTR2 expression or release of an antibody against SSAs [25].

Most primary NENs express SSTR2, in particular, isoform A. Locoregional and distant metastases often maintain the same level of SSTR2 expression. However, it seems that other SSTRs, such as SSTR5, may be overexpressed in metastatic disease. Immunohistochemistry staining performed on well-differentiated GEP-NENs showed that the expression of SSTR5 was higher in distant metastases in comparison to their primary site. Moreover, tumours showed intra-tumour heterogeneity for both receptors [32]. It seems that only the expression of SSTR2 has a prognostic value in NENs receiving SSAs. Nevertheless it may be worth assessing expression of both SSTR2 and SSTR5, especially in the metastatic setting, regardless of the primary tumour status to evaluate response to SSAs and to better select patients for treatment [32,50,85,162].

## 7. Cell Signalling Pathway Heterogeneity: The mTOR Pathway

In NENs, several pathways play a major role in cancer growth and metastasis, and have recently been explored as possible treatment targets in advanced cases [163]. It is not always easy to identify which signalling pathways are involved in a specific case, especially because inter-tumour heterogeneity is often present between the primary tumour and the metastatic sites [164].

The PI3K/protein kinase B (AKT)/mTOR_signalling pathway plays a fundamental role in the regulation of cell growth and proliferation, angiogenesis, nutrient availability, metabolism, and apoptosis [165]. The mTOR signalling pathway also plays a crucial role in the tumour microenvironment, and in the interaction between cancer cells and the tumour microenvironment, where it regulates angiogenesis, metabolism and modulates the immune response [166]. mTOR activation is a common feature of both primary and metastatic GEP-NENs suggesting that it is involved in cancer development and progression [167,168]. mTOR activation forms two complexes, mTORC1 that mainly regulates cell growth and metabolism, and mTORC2, which principally controls cell proliferation and survival [169]. Alteration of this pathway has been reported in several cancers. Many compounds that inhibit PI3K, AKT and mTOR have therefore been investigated and developed, including rapamycin and its derivatives [170]. Rapamycin inhibits the mTORC1 complex, while mTORC2 is not sensitive to acute treatment with rapamycin [171]. Everolimus, a derivative of rapamycin, is an established therapeutic option in selected NENs [49,172]. A phase three randomised clinical trial (RADIANT-3) demonstrated that patients with advanced pancreatic NENs who have been treated with everolimus had a significantly better progression-free survival and achieved a more stable disease in comparison with patients receiving a placebo only [173]. Similar results were seen in another phase three randomised clinical trial where sunitinib, a pan-tyrosine kinase inhibitor, was administered in patients with advanced pancreatic NEN [174]. Currently, everolimus and sunitinib are recommended for progressive and metastatic well to moderately differentiated pancreatic NENs after failure of SSAs or chemotherapy, or as a first-line option when SSAs or chemotherapy are not feasible [158]. Everolimus can also be used in intestinal NENs after failure of SSA or PRRT. These oral drugs are generally well tolerated; however, selected food interactions associated with the inhibition of the P450 (CYP) 3A pathway may lead to significant toxicity [175]

Unfortunately, resistance to mTOR inhibitors is a common phenomenon and disease stabilization is mainly responsible for the improvement in progressive-free survival There are several possible mechanisms for rapamycin resistance and treatment failure, with tumour heterogeneity being one of the possible contributing factors. Spatial heterogeneous alterations in the mTOR pathway could affect response to treatment, as the coexistence of different mutations could lead to rapamycin resistance [176].

There are only few studies available that have investigated tumour heterogeneity in the mTOR pathway in GEP-NENs (Table 2).

In a study of 27 cases of synchronous well-differentiated grade 1 metastatic SI-NETs, it was shown that although mTOR activation was present in most of the cases, in the primary and the metastatic sites (liver and loco-regional lymph nodes), a difference in the activation and regulation of this pathway was noted between the primary and metastatic sites. In particular, more than 95% of the liver metastases showed a higher activation of this signalling pathway with overexpression of different components of the pathway [32]. A molecular profiling study of liver metastases from SI-NENs showed that the mTOR signalling pathway was overexpressed in liver metastases from SI-NENs in comparison to the primary tumour, demonstrating that during the metastatic process there is progressive epigenetic dysregulation of this pathway [43]. Similarly, another study on 43 resected pancreatic NENs showed that liver metastases showed overexpression of the mTOR pathway in comparison to the primary tumour [177]. High mTOR expression is a prognostic factor and is linked to a worse prognosis in pancreatic NENs [179]. Higher expression of phospho-mTOR, the activated form of the mTOR, has been shown to correlate with liver recurrence and a worse survival in patients undergoing radical surgery for pancreatic NENs [180]. In contrast, in other studies of NENs from different origins, including SI, mTOR was significantly higher in primary lesions than in metastases, suggesting that further progression of the disease may be linked to other genetic alterations [49,178]. Although the evidence is limited, it also seems that its inactivation is a marker of aggressive disease in both primary and metastatic sites, since progression to a less differentiated tumour has been noted in mTOR negative primary tumours as well as in metastatic disease, indicating a possible change over time [32,178]. Indeed, it has been shown that mTOR negative patients treated with everolimus have worse outcomes, as the activation of the mTOR pathway, which was more frequent in ileal NENs, was linked to a better progression-free survival and overall survival in comparison with negative cases [181]. It is not clear whether there is a link between alteration of the mTOR pathway and the site of origin of the primary tumour. On one hand, Catena et al. found that 80% of poorly differentiated NECs showed mTOR expression and that there was no correlation with the site of origin, although most of the cases were pancreatic NECs [168]. On the other hand, Kasajima et al. demonstrated that mTOR expression and activity was higher in foregut (i.e., upper gastrointestinal and pancreatic) NENs in comparison with midgut (i.e., ileum and proximal large bowel) NENs, and especially in metastatic foregut NENs. This suggests that there may be a difference in the activity pattern of the mTOR pathway depending on the location of the primary tumour and stage that may influence the efficacy of the mTOR inhibitors [182]. Although SI-NENs also show abnormal activation of the mTOR pathway, and aberrant activation of the mTOR pathway is a common event in NENs, especially in more advanced cases, it is plausible that heterogeneous expression of this pathway may be more relevant than is currently known and that this heterogeneity must be taken into account and further explored to optimize results from targeted therapies in NENs [183,184,185].

## 8. Present and Future Strategies

One of the major issues of targeted therapy is the development of treatment resistance, as cancer cells are able to adapt and resist under selective treatment pressure. The presence within the tumour of subpopulations of cancer cells that are able to escape immune surveillance and evolve over time accounts for the development of treatment resistance and disease progression [9].

Despite the effectiveness of different treatment options for NENs, relapse and progression are common, and up-to-date strategies to address NEN heterogeneity are needed. The approaches to tackle spatial and temporal heterogeneity can be directed toward either identifying predictive and prognostic biomarkers for treatment success and/or designing optimal treatment sequencing and combination to minimize relapse and progression.

In the era of personalized medicine, attention is now focused on finding alternative methods to biopsy that are reliable, minimally invasive, easy to perform and repeatable over the course of the disease. Several strategies have been developed. For NENs, there is preliminary evidence for the usefulness of several techniques, but further studies are needed to confirm whether these techniques have a role to play.

Liquid biopsies have been proposed as a less invasive alternative to tissue biopsy that also allows identification of minimal residual disease and the detection of resistance mechanisms. Moreover, liquid biopsies could be a valuable tool in detecting inter-tumour heterogeneity in circulation cancer cell clones [116]. For NENs, liquid biopsy seems to be highly accurate for diagnosis and in assessing grade, stage and progression [186]. The NETest is the only novel biomarker that has been validated as reliable tool to assess the effectiveness of treatment and predict recurrence [187,188]. In particular, the predictive Quotient (PPQ), which integrates NETest gene transcripts with the tissue Ki-67 index seems to effectively predict the response to PRRT [189].

Single-cell RNA sequencing has been used in different cancers to identify intra-tumoural cell heterogeneity that could explain relapse and recurrence after treatment [190]. Its potential use in NENs has been shown in pancreatic NENs where the spatiotemporal heterogeneity of cancer cells in subpopulations in the primary tumour and metastatic sites has been demonstrated [191].

Advanced image analysis (radiomics) has enabled the extraction of accurate quantitative information on spatial and longitudinal heterogeneity of tumour biology from routinely acquired tumour imaging [192]. This method allows visual assessment and mapping of the whole tumour and its microenvironment in order to non-invasively characterise the spatial and temporal heterogeneity of the tumour. This information is often not visible to the naked eye, and can be used as a predictor of disease progression and patient survival [193,194,195]. Although the available data on the application of radiomics in NENs are still limited, it has been shown that radiomics features could be useful not only to assess tumour grade, but also as a biomarker to predict and evaluate the response to treatment as it allows the identification of inter-patient tumour heterogeneity [52,64,196,197,198]. The assessment of tumour heterogeneity seems to provide more accurate information in comparison to standard imaging parameters and may enable implementation of optimal individualized treatment [199].

The evaluation of tumour heterogeneity could also be particularly useful in guiding treatment selection at diagnosis and following recurrence, as outcomes could be improved by targeting different components of the disease. The choice of subsequent treatment should be based on tumour biology at the time of recurrence or progression [200,201]. For pancreatic NENs, where outcomes are generally favourable but relapse common in advanced cases, tailored treatment sequencing strategies could help in selecting the most effective treatment option for each particular case [202]. Knowledge of spatiotemporal heterogeneity therefore represents the prerequisite for a tailored approach.

## 9. Conclusions

The evidence on NENs heterogeneity has grown significantly in the last decade, though much still needs to be investigated. The link between spatial and temporal tumour heterogeneity in the expression of targets and patient outcomes has been highlighted, showing how this information needs to be considered for a more individualized treatment strategy. A single standard fixed diagnostic algorithm may not work for this highly heterogeneous group of tumours. Individualized strategies in NEN diagnosis can lead to improved outcomes, better selection of candidates for specific treatment and evaluation in the post-treatment setting. Single tumour biopsies seem to be inadequate for characterizing the whole landscape of alterations of NENs, as they may failure to capture the real behaviour of the disease. Metastatic lesions are often discordant from the primary tumour as they often show features that were not present in the primary tumour. Temporal heterogeneity is also a consequence of treatment selective pressure and repeat biopsies should be considered at the time of initial recurrence or disease progression. On a practical level, however, extensive and repeat investigations may not be feasible with regards to safety and costs. The increase in the amount of information available from tumour biology will eventually create many challenges in the clinical setting. Future research should therefore also focus on identifying new modalities and relevant markers that can simplify the treatment decision-making process and optimize treatment.

## Figures and Tables

**Table 1:** Classification for gastroenteropancreatic neuroendocrine neoplasms (GEP-NENs).

Terminology	Differentiation	Grade	Mitotic Rate (Mitoses/2 mm^2^) *	Ki-67 Index % **
NET, G1	Well differentiated	Low	<2	<3%
NET, G2		Intermediate	2–20	3–20%
NET, G3		High	>20	>20%
NEC, small-cell type (SCNEC)	Poorly differentiated	High	>20	>20%
NEC, large-cell type (LCNEC)			>20	>20%
MiNEN	Well or poorly differentiated	Variable	Variable	Variable

* mitotic rates are determined by counting in 50 fields of 0.2 mm^2^; ** Ki-67 values are expressed as the percentage of positive cells. Abbreviations: LCNEC, large-cell neuroendocrine carcinoma; MiNEN, mixed neuroendocrine–non-neuroendocrine neoplasm; NEC, neuroendocrine carcinoma; NET, neuroendocrine tumour; SCNEC, small-cell neuroendocrine carcinoma.

**Table 2:** mTOR pathway alterations in gastroenteropancreatic neuroendocrine neoplasms (GEP-NENs).

Authors	Site	mTOR	Other Findings
Tran et al. [177]	Metastatic (nodal and liver) pancreatic	↑	↓SSTR
Borga et al. [32]	Metastatic (nodal and liver) ileal	↑ (++ liver)	↑SSTR5 (liver) intra-spot SSTR2A/SSTR5 expression heterogeneity
Karphatakis et al. [43]	Metastatic (liver) small intestine	↑	-
Gilbert et al. [178]	NENs (including small intestine)	↑primary ↓metastases	-

Table 2 summarises the studies that have investigated the pattern of mTOR expression in primary and metastatic GEP-NENs. Two studies have also investigated the SSTR expression in both sites. Abbreviations; mTOR: mammalian target of rapamycin; SSTR: somatostatin receptor.

## Abbreviations

NENs: neuroendocrine neoplasms; TME: tumour microenvironment; SSRTs: Somatostatin receptors; SSTR2: Somatostatin receptor type 2; mTOR: Mammalian target of rapamycin; MDSC: myeloid-derived suppressor cells; TAM: tumour-associated macrophages; TAN: tumour-associated neutrophils; DC: dendritic cells; 68Ga-DOTA-peptides PECT/CT: Gallium-68 DOTA peptides positron emission tomography/computer tomography (PET/CT); 18F-FDG: flourine-18 fluorodeoxyglucose; PRRT: peptide receptor radionuclide therapy.
